# Understanding COVID-19 vaccine demand and hesitancy: A nationwide online survey in China

**DOI:** 10.1371/journal.pntd.0008961

**Published:** 2020-12-17

**Authors:** Yulan Lin, Zhijian Hu, Qinjian Zhao, Haridah Alias, Mahmoud Danaee, Li Ping Wong

**Affiliations:** 1 Department of Epidemiology and Health Statistics, School of Public Health, Fujian Medical University, Fuzhou, Fujian, China; 2 State Key Laboratory of Molecular Vaccinology and Molecular Diagnostics, National Institute of Diagnostics and Vaccine Development in Infectious Diseases, School of Public Health, Xiamen University, Xiamen, Fujian, China; 3 Centre for Epidemiology and Evidence-Based Practice, Department of Social and Preventive Medicine, Faculty of Medicine, University of Malaya, Kuala Lumpur, Malaysia; University of Pittsburgh, UNITED STATES

## Abstract

**Background:**

This study attempts to understand coronavirus disease 2019 (COVID-19) vaccine demand and hesitancy by assessing the public’s vaccination intention and willingness-to-pay (WTP). Confidence in COVID-19 vaccines produced in China and preference for domestically-made or foreign-made vaccines was also investigated.

**Methods:**

A nationwide cross-sectional, self-administered online survey was conducted on 1–19 May 2020. The health belief model (HBM) was used as a theoretical framework for understanding COVID-19 vaccination intent and WTP.

**Results:**

A total of 3,541 complete responses were received. The majority reported a *probably yes* intent (54.6%), followed by a *definite yes* intent (28.7%). The perception that vaccination decreases the chances of getting COVID-19 under the perceived benefit construct (OR = 3.14, 95% CI 2.05–4.83) and not being concerned about the efficacy of new COVID-19 vaccines under the perceived barriers construct (OR = 1.65, 95% CI 1.31–2.09) were found to have the highest significant odds of a definite intention to take the COVID-19 vaccine. The median (interquartile range [IQR]) of WTP for COVID-19 vaccine was CNY¥200/US$28 (IQR CNY¥100–500/USD$14–72). The highest marginal WTP for the vaccine was influenced by socio-economic factors. The majority were *confident* (48.7%) and *completely confident* (46.1%) in domestically-made COVID-19 vaccine. 64.2% reported a preference for a domestically-made over foreign-made COVID-19 vaccine.

**Conclusions:**

The findings demonstrate the utility of HBM constructs in understanding COVID-19 vaccination intent and WTP. It is important to improve health promotion and reduce the barriers to COVID-19 vaccination.

## Author summary

This study investigated vaccine demand and hesitancy by assessing the intention to vaccinate against COVID-19 and willingness-to-pay. We found that a considerable proportion of the public in China has a definite intention to receive the COVID-19 vaccine; a higher proportion expressed a probable intention. Perceived benefits and barriers to vaccination (namely vaccine efficacy and adverse event concerns) of the health belief model constructs were significant predictors of COVID-19 vaccination intent. A substantial proportion was concerned about fake or faulty COVID-19 vaccines; however, this was not a significant predictor of vaccination intention. The willingness-to-pay for the COVID-19 was determined and was found to be positively associated with income. Higher confidence in domestically-made COVID-19 vaccines was also found in this study. The preference for domestically-made over foreign-made COVID-19 vaccines indicates that a future COVID-19 vaccine developed by domestic companies will receive a favourable response from the public in China.The findings of this study provide useful guidance for tailored interventions to enhance the acceptance of a new COVID-19 vaccine once it is available. Promotional messages framing the benefit of vaccination and concerns about new vaccine safety to enhance vaccine uptake is warranted.

## Introduction

The origin of the outbreak of coronavirus disease 2019 (COVID-19) caused by severe acute respiratory syndrome coronavirus 2 (SARS-CoV-2) was initially detected in Wuhan, China in December 2019. The new coronavirus spread rapidly around the world within a month of its onset. On 11 March 2020, the World Health Organization (WHO) declared COVID-19 a worldwide pandemic. By the end of May, the COVID-19 had infected over 5 million people across 215 countries or territories and caused more than 300,000 fatalities worldwide [1].

In the absence of a vaccine or effective treatment, all the nations worldwide are struggling to contain the spread of the COVID-19 with the enforcement of quarantine and lockdowns, social distancing measures, community-use of facemasks at all times, and travel restrictions. These have resulted in the tremendous impairment of physical and psychosocial well-being of people and has driven a massive decline in the global economy. The multi-faceted catastrophic consequences associated with the COVID-19 outbreak have intensified international efforts in developing an effective prevention method to keep outbreaks under control. There is an intense international effort in developing a safe and effective COVID-19 vaccine, with an estimate of over 100 candidate vaccines currently in different development stages [2], and several candidate vaccines already in clinical trials [3]. A vaccine against COVID-19 may soon be available for public use; as such, urgent understanding is warranted to investigate the acceptability of a COVID-19 vaccine to prepare for effective promotion strategies.

Intention to be vaccinated against an infectious disease is recognized as a foremost issue affecting the success of vaccination programs. In China, vaccine hesitancy is a complex public health issue. In the last decade, vaccine scandals and a series of reports about the serious side-effects of vaccination have increased vaccination hesitancy and distrust in the country’s immunization program [4]. There are multiple factors influencing vaccination intention. The health belief model (HBM), which explains and predicts a variety of human behaviors, is one of the most commonly used models to determine vaccination intention [5]. HBM constructs have been recognized as an important predictor of influenza vaccination uptake in previous studies [6–8]. The HBM comprises several main constructs: perceived susceptibility, severity, benefits, barriers, self-efficacy to engage in a behaviour and cues to action [9]. Perceived susceptibility refers to beliefs regarding vulnerability to infection, while perceived severity refers to beliefs regarding the negative effects of contracting the infection. In relation to vaccination, perceived benefits are defined as an individual’s beliefs about being vaccinated, and perceived barriers are described as the belief that being vaccinated is restricted due to psychosocial, physical or financial factors. Cues to action include information, people and events that guide an individual to be vaccinated [9,10].

Willingness-to-pay (WTP) refers to the maximum amount in monetary terms that an individual would be willing to sacrifice to obtain the benefits of a program, service or health technology [11]. In vaccination decisions, the decision to vaccinate depends on the WTP of an individual for a vaccination to obtain increased health benefits [12]. HBM constructs have also been used to explain WTP for influenza vaccination [13]. More evidence about public acceptance and the WTP for the COVID-19 vaccine is essential to evaluate the feasibility of the implementation of vaccination programme in China when the vaccine is available and also to provide insights into future pricing considerations, demand forecasts, and the implementation of the national COVID-19 immunization program.

Therefore, the objectives of this study were to determine COVID-19 vaccination intent and WTP. The HBM was used to predict vaccination intention and the highest marginal WTP. Other influences of vaccination intention and WTP, such as demographic factors and psychological characteristics (namely participants’ health perception, presence of chronic diseases, and knowing someone in the community who has had COVID-19) assumed to exert their effect via changes in the components of the HBM were also investigated.

Recently, distrust in the country’s immunization program and domestically-made vaccines has not only resulted in vaccine hesitancy but also caused an increasing number of people favoring imported vaccines or seeking vaccinations abroad [4,14,15]. Considering that there will be vaccines developed by Chinese manufacturers as well as from other countries, there have been concerns that the public in China is more inclined to choose a foreign-made vaccine over vaccines produced in China [16]. Therefore, this study also attempted to explore vaccine confidence in domestically-made COVID-19 vaccines and vaccine preference.

## Methods

### Ethics statement

This study was approved by the Medical Ethics Committee at the Fujian Medical University, Fuzhou, China (Approval: FJMU 2020 NO.1). Respondents were informed that their participation was voluntary, and consent was implied on the completion of the questionnaire.

### Study participants and survey design

We commenced a cross-sectional, web-based anonymous survey using an online questionnaire. The survey was conducted from 1–19 May 2020. The research team used WeChat (the most popular social media platform in China) to advertise and circulate the survey link to their network members. Network members were requested to distribute the survey invitation to all their contacts throughout the country. The participants were informed that their participation was voluntary, and consent was implied through their completion of the questionnaire. The inclusion criteria were that the respondents were Chinese citizens who were at least 18 years old, and able to comprehend and read Chinese.

### Instruments

The survey consisted of questions that assessed 1) demographic background, self-perceived health status, and COVID-19 experience; 2) perception of COVID-19 and COVID-19 vaccination; 3) intention to receive a COVID-19 vaccine; 4) WTP for a COVID-19 vaccine; and 5) vaccine confidence and preference.

#### Demographics, health status and COVID-19 experience

Personal details, including age, gender, ethnicity, religion, marital status, occupation and average monthly household income were collected. The participants were also queried if they had existing chronic diseases and to rate their overall health status. COVID-19 experience assessed whether participants had any family members, or any friends, neighbors or colleagues with confirmed COVID-19.

#### Perception of COVID-19 and COVID-19 vaccination

HBM-derived items were used to measure the participants’ perception of COVID-19 and COVID-19 vaccination [17,18]. The questions probed perceived susceptibility to COVID-19 (three items), perceived severity of COVID-19 (three items), perceived benefits of a COVID-19 vaccine (two items), perceived barriers to getting a vaccination against COVID-19 (five items) and cues to action (two items). All the response options were ‘strongly agree’, ‘agree’, ‘disagree’ or ‘strongly disagree’.

#### Intention to receive a COVID-19 vaccine and willingness to pay (WTP)

The intention to accept a COVID-19 vaccine was measured using a one-item question (If a vaccine against COVID-19 was available on the market, would you take it?) on a four-point scale (‘definitely not’ to ‘definitely yes’). WTP was measured using a one-item question (What is the maximum amount you are willing to pay for the COVID-19 vaccine?) on a nine-point scale (CNY¥100/US$14 to CNY¥900/US$125, at a currency ratio of 7:1). The price range options were based on the approximate minimum-maximum price range of currently available vaccines in China.

#### Vaccine confidence and preference

Participants were asked to rate their level of confidence in using 1) domestically-manufactured and 2) foreign-manufactured COVID-19 vaccine on a four-point rating scale (‘completely confident’, ‘confident’, ‘not confident’, and ‘completely not confident’). Preference in domestic or foreign/imported COVID-19 vaccine was also queried. The full questionnaire is provided as supplemental material (S1 Text).

### Statistical analysis

We ran univariate analyses followed by a multivariable logistic regression analysis, including all factors showing significance (p<0.05), to determine factors associated with the definite intention to take the COVID-19 vaccine. Odds ratios (OR), 95% confidence intervals (95% CI) and *p*-values were calculated for each independent variable [19]. The model fit of multivariable logistic regression analysis was assessed using the Hosmer-Lemeshow goodness-of-fit test [20]. The distribution of WTP responses does not follow a normal distribution. The majority of respondents were willing to pay between CNY¥100 and CNY¥400, and a lower proportion reported a WTP of CNY¥500 and above. Hence the WTP was divided into three price ranges (CNY¥100/200, CNY¥300/400, and ≥CNY¥500). A multivariable multinomial logistic regression was employed to model factors associated with marginal WTP for the COVID-19 vaccine for three price ranges, with CNY¥100/200, the lowest coded category as the reference group. Likewise, only significant factors in the univariate analyses, with *p*-values of <0.05, were selected for the multinomial logistic regression analysis. The possibility of income level as a moderator on the relationship between the constructs of HBM (scores of perceived susceptibility, severity, benefit, barriers and cues to action) and WTP was investigated using Hayes’ PROCESS macro version 3.5 [21]. All statistical analyses were performed using the Statistical Package for the Social Sciences version 20.0 (IBM Corp., Armonk, NY, USA). A *p*-value of less than 0.05 was considered statistically significant.

## Results

### Demographics

**Fig 1:**
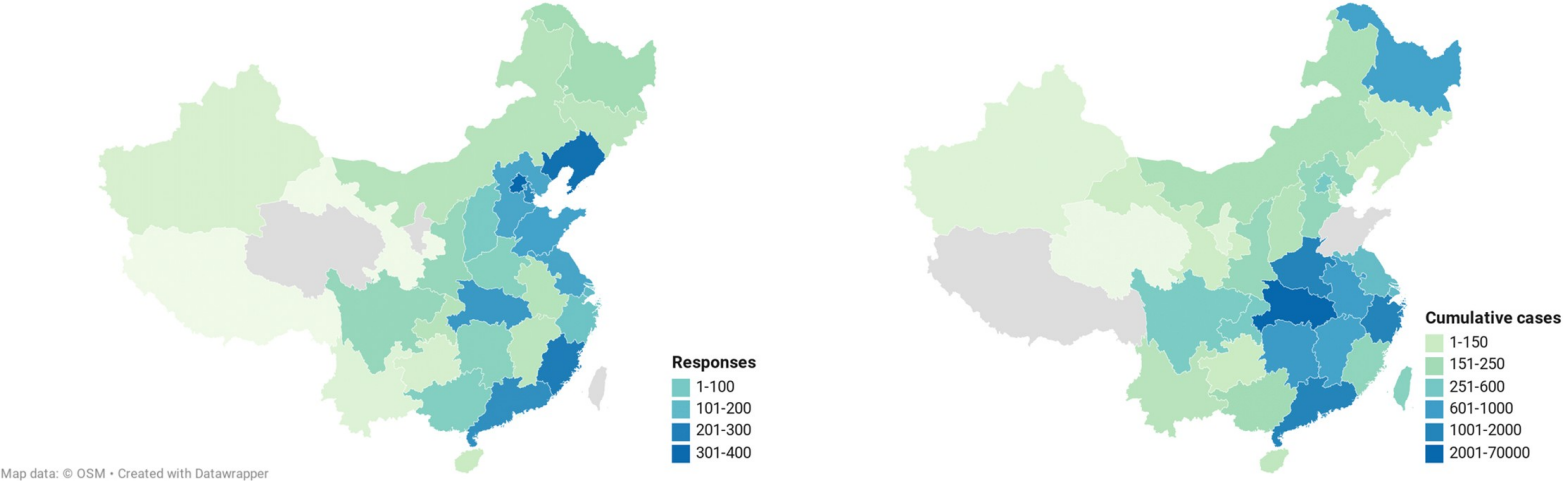
Geographical distribution of responses of the study and confirmed cases of COVID-19 as of 22^nd^ May 2020.

A total of 3,541 complete responses were received. Table 1 shows the demographics of our study participants compared with the general adults population in China [22,23]. The study received responses from participants of all regions in mainland China and of diverse demographics, as shown in the overall column in Table 2. Of note, the study participants had a higher representation of participants aged 26 to 35 years old (47.2%). The great majority had a college degree (64.8%) compared to secondary school and below (35.2%). A higher proportion of participants from urban (84.6%) than rural (15.4%) localities responded to our survey. Near half of the study participants were of professional and managerial occupations (49.9%). The study has less representation of participants from the western and central regions. Fig 1 shows the mapping of the geographical distribution of responses and the regional total number of confirmed cases of COVID-19 [24]. The study has slight over-representation of responses from the Fujian province, where the study originates. High responses from Hubei and Guanhzhou provinces in this study correspond to the high number of confirmed cases in these provinces.

**Table 1:** Comparison of demographic characteristics of the study population and the general adults population in China, 2010.

Characteristics	N	% Study population, N = 3541	% Total population, N = 933785258^†^
Age group (years)			
18–25	642	18.1	13.6^†^
26–35	1673	47.2	21.2^†^
36–45	860	24.3	26.0^†^
46–70	366	10.3	39.1^†^
Gender			
Male	1702	48.1	50.8
Female	1839	51.9	49.2
Average annually household income (CNY) ^‡^			
<50000	841	23.8	39.0
50001–120,000	1341	37.9	42.9
>120000	1359	38.4	18.1
Region^¶^			
East China	1191	33.6	29.5
South China	417	11.8	16.8
West China	164	4.6	21.8
North China	1460	41.2	20.6
Central China	309	8.7	11.3

^†^ Total number of adults 20 to 70 years of age according to China national census in 2010 [22].

^‡^ China household income distribution: Urban [23].

^¶^ Northern region (Beijing, Tianjian, Hebei, Shanxi, Inner Mogolia, Liaoning, Jilin); Central (Henan, Hubei); East region (Shanghai, Jiangsu, Zhejiang, Anhui, Fujian, Jiangxi, Shandong, Taiwan); Southern region (Hunan, Guangdong, Guangxi, Hainan, Hong Kong, Macao); Western region (Chongqing, Sichuan, Guizhou, Yunnan, Tibet, Shannxi, Gansu, Qinghai, Ninxia, Xinjiang).

**Table 2:** Demographics characteristics and factors associated with a definite intention to take the COVID-19 vaccine (N = 3541).

		Univariable analysis				Multivariable logistic regression	
	Overal l N(%)	Definitely yes n = 1018	Probably yes /Probably no/Definitely no n = 2523	Unadjusted OR (95% CI)	*p*-value	Definitely yes vs. Probably yes/Probably no/Definitely no Adjusted OR (95% CI)	*p*-value
*Demographics*							
Age group (years)							
18–25	642 (18.1)	157 (24.5)	485 (75.5)	Reference			
26–35	1673 (47.2)	496 (29.6)	1177 (70.4)	1.30 (1.06–1.60)	0.061		
36–45	860 (24.3)	252 (29.3)	608 (70.7)	1.28 (1.02–1.62)			
46–70	366 (10.3)	113 (30.9)	253 (69.1)	1.38 (1.04–1.84)			
Gender							
Male	1702 (48.1)	469 (27.6)	1233 (72.4)	Reference	0.137		
Female	1839 (51.9)	549 (29.9)	1290 (70.1)	1.12 (0.97–1.30)			
Marital status							
Married	2770 (78.2)	831 (30.0)	1939 (70.0)	1.34 (1.11–1.61)	0.002	1.21 (0.98–1.50)	0.082
Single	771 (21.8)	187 (24.3)	584 (75.7)	Reference		Reference	
Highest education level							
High school and below	1248 (35.2)	359 (28.8)	889 (71.2)	1.00 (0.86–1.16)	1.000		
Tertiary	2293 (64.8)	659 (28.7)	1634 (71.3)	Reference			
Occupation category							
Professionals and managerial	1591 (49.9)	428 (26.9)	1163 (73.1)	1.29 (0.91–1.83)		1.25 (0.84–1.87)	0.276
Industrial workers	423 (11.9)	121 (28.6)	302 (71.4)	1.41 (0.95–2.08)		1.27 (0.81–1.99)	0.292
Self-employed	416 (11.7)	136 (32.7)	280 (67.3)	1.71 (1.16–2.52)	0.003	1.66 (1.07–2.59)	0.025
Farmers	127 (3.6)	34 (26.8)	93 (73.2)	1.28 (0.77–2.15)		1.21 (0.68–2.15)	0.526
Service personnel	413 (10.7)	147 (35.6)	266 (64.4)	1.94 (1.32–2.86)		1.61 (1.05–2.49)	0.030
Housewife/ Retiree/ Unemployed	368 (10.4)	107 (29.1)	261 (70.9)	1.44 (0.97–2.15)		1.25 (0.79–1.98)	0.344
Student	203 (5.7)	45 (22.2)	158 (77.8)	Reference		Reference	
Average annual household income (CNY¥)							
<50000	841 (23.8)	256 (30.4)	585 (69.6)	1.01 (0.82–1.26)			
50001–120000	1341 (37.9)	377 (28.1)	964 (71.9)	0.91 (0.74–1.11)	0.262		
120001–170000	646 (18.2)	170 (26.3)	476 (73.7)	0.83 (0.65–1.05)			
>170000	713 (20.1)	215 (30.2)	498 (69.8)	Reference			
Location							
Urban	2997 (84.6)	850 (28.4)	2147 (71.6)	1.13 (0.93–1.38)	0.237		
Rural	544 (15.4)	168 (30.9)	376 (69.1)	Reference			
Current location							
East	1191 (33.6)	333 (28.0)	858 (72.0)	Reference		Reference	
South	417 (11.8)	135 (32.4)	282 (67.6)	2.09 (1.38–3.19)		1.24 (0.96–1.600	0.096
West	164 (4.6)	41 (25.0)	123 (75.0)	1.44 (0.95–2.16)	p<0.001	0.87 (0.59–1.29)	0.487
North	1460 (41.2)	382 (26.2)	1078 (73.8)	1.16 (0.80–170)		0.94 (0.78–1.14)	0.533
Central	309 (8.7)	127 (41.1)	182 (58.9)	1.06 (0.73–1.54)		1.67 (1.26–2.18)	p<0.001
*Experience with COVID-19*							
Ever have experience with COVID-19							
No	3301 (93.2)	953 (28.9)	2348 (71.1)	1.09 (0.81–1.47)	0.605		
Yes	240 (6.8)	65 (27.1)	175 (72.9)	Reference			
*Health characteristics*							
Ever diagnosed with chronic diseases							
Yes	220 (6.2)	55 (25.0)	165 (75.0)	Reference	0.219		
No	3321 (93.8)	963 (29.0)	2358 (71.0)	1.23 (0.90–1.68)			
Perceived overall health							
Very good	1142 (32.3)	397 (34.8)	745 (65.2)	1.57 (1.32–1.87)		1.74 (1.44–2.09)	p<0.001
Good	1132 (32.0)	300 (26.5)	832 (73.5)	1.06 (0.89–1.28)	p<0.001	1.08 (0.89–1.31)	0.423
Fair/Poor/Very poor	1267 (35.8)	321 (25.3)	946 (74.7)	Reference		Reference	
***Health belief***							
**Perceived susceptibility**							
Chance of getting COVID-19 in the next few months is great							
Strongly agree/agree	1107 (31.3)	271 (24.5)	836 (75.5)	Reference	p<0.001	Reference	
Disagree/strongly disagree	2434 (68.7)	747 (30.7)	1687 (69.3)	1.37 (1.16–1.61)		1.01 (0.813–1.25)	0.939
Worry about the likelihood of getting COVID 19							
Strongly agree/agree	1528 (43.2)	425 (27.8)	1103 (72.2)	Reference	0.294		
Disagree/strongly disagree	2013 (56.8)	593 (29.5)	1420 (70.5)	1.08 (0.94–1.26)			
Getting COVID-19 is currently a possibility for me							
Strongly agree/agree	990 (28.0)	235 (23.7)	755 (76.3)	Reference	p<0.001	Reference	
Disagree/strongly disagree	2551 (72.0)	783 (30.7)	1768 (69.3)	1.42 (1.20–1.69)		1.26 (1.01–1.56)	0.043
**Perceived severity**							
Complications from COVID-19 are serious							
Strongly agree/agree	3065 (86.6)	897 (29.3)	2168 (70.7)	1.21 (0.97–1.51)			
Disagree/strongly disagree	476 (13.4)	121 (25.4)	355 (74.6)	Reference	0.091		
I will be very sick if I get COVID-19							
Strongly agree/agree	2171 (61.3)	627 (28.9)	1544 (71.1)	1.02 (0.88–1.18)	0.849		
Disagree/strongly disagree	1370 (38.7)	391 (28.5)	979 (71.5)	Reference			
I am afraid of getting COVID-19							
Strongly agree/agree	2984 (84.3)	879 (29.5)	2105 (70.5)	1.26 (1.02–1.55)	0.032	1.46 (1.14–1.87)	0.003
Disagree/strongly disagree	557 (15.7)	139 (25.0)	418 (75.0)	Reference		Reference	
**Perceived benefits**							
Vaccination is a good idea because it makes me feel less worried about catching COVID-19							
Strongly agree/agree	3089 (87.2)	926 (30.0)	2163 (70.0)	1.68 (1.32–2.13)	p<0.001	1.39 (1.05–1.84)	0.022
Disagree/strongly disagree	452 (12.8)	92 (20.4)	360 (79.6)	Reference		Reference	
Vaccination decreases my chance of getting COVID-19 or its complications							
Strongly agree/agree	3277 (92.5)	981 (29.9)	2296 (70.1)	2.62 (1.84–3.74)		3.14 (2.05–4.83)	p<0.001
Disagree/strongly disagree	264 (7.5)	37 (14.0)	227 (86.0)	Reference	p<0.001	Reference	
**Perceived barriers**							
Worry the possible side-effects of COVID-19 vaccination would interfere with my usual activities							
Strongly agree/agree	2425 (68.5)	581 (24.0)	1844 (76.0)	Reference		Reference	
Disagree/strongly disagree	1116 (31.5)	437 (39.2)	679 (60.8)	2.04 (1.75–2.38)	p<0.001	1.52 (1.25–1.84	p<0.001
Concern about the efficacy of the COVID-19 vaccination							
Strongly agree/agree	2520 (71.2)	600 (23.8)	1920 (76.2)	Reference	p<0.001	Reference	
Disagree/strongly disagree	1021 (28.8)	418 (40.9)	603 (59.1)	2.22 (1.90–2.59)		1.65 (1.31–2.09)	p<0.001
Concern about the safety of the COVID-19 vaccination							
Strongly agree/agree	2569 (72.6)	632 (24.6)	1937 (75.4)	Reference	p<0.001	Reference	
Disagree/strongly disagree	972 (27.4)	386 (39.7)	586 (60.3)	2.02 (1.73–2.36)		1.21 (0.95–1.53)	0.123
Concern of my affordability (high cost) of getting the COVID-19 vaccination							
Agree	2658 (75.1)	720 (27.1)	1938 (72.9)	Reference	p<0.001	Reference	
Disagree	883 (24.9)	298 (33.7)	585 (66.3)	1.37 (1.16–1.62)		0.97 (0.79–1.18)	0.745
Concern if the faulty/fake COVID-19 vaccine							
Strongly agree/agree	2902 (82.0)	803 (27.7)	2099 (72.3)	Reference	0.003	Reference	
Disagree/strongly disagree	639 (18.0)	215 (33.6)	424 (66.4)	1.33 (1.10–1.59)		0.94 (0.74–1.18)	0.582
**Cues to action**							
I will only take the COVID-19 vaccine if I was given adequate information about it							
Strongly agree/agree	3260 (92.1)	937 (28.7)	2323 (71.3)	Reference	1.000		
Disagree/strongly disagree	281 (7.9)	81 (28.8)	200 (71.2)	1.00 (0.77–1.31)			
I will only take the COVID-19 vaccine if the vaccine is taken by many in the public							
Agree	2914 (82.3)	754 (25.9)	2160 (74.1)	Reference		Reference	
Disagree	627 (17.7)	264 (42.1)	363 (57.9)	2.08 (1.74–2.49)	p<0.001	1.88 (1.52–2.33)	p<0.001

Hosmer–Lemeshow test, chi-square: 2.275, p-value: 0.971; Nagelkerke R^2^: 0.127.

### Health beliefs

The participants had low perceptions of susceptibility. The majority disagreed that there was a great chance of getting COVID-19 in the next few months (68.7%), were also not worried about the likelihood of getting COVID-19 (56.8%), and disagreed that it was currently possible that they would get COVID-19 (72.0%). The participants had high perceptions of the severity of COVID-19; 86.6% agreed that the complications of COVID-19 are serious and the vast majority were afraid of getting COVID-19 (84.3%). High perceptions of the benefits of COVID-19 vaccination were reported. The majority (87.2%) perceived the benefit of feeling less worry of contracting the coronavirus after getting the vaccine. A higher proportion (92.5%) perceived benefit of the COVID-19 vaccine in reducing the risk of infection and resultant complications. Under the perceived barriers construct, concerns about the COVID-19 vaccine being faulty/fake and affordability were reported by 82.0% and 75.1%, respectively. Concerns about safety (72.6%) and efficacy (71.2%) were also notable. Many of the participants (92.1%) reported that they would only receive the COVID-19 vaccine if given adequate information, and 82.3% reported that they will only take the vaccine if taken by many in the public.

### COVID-19 vaccination intent

**Fig 2:**
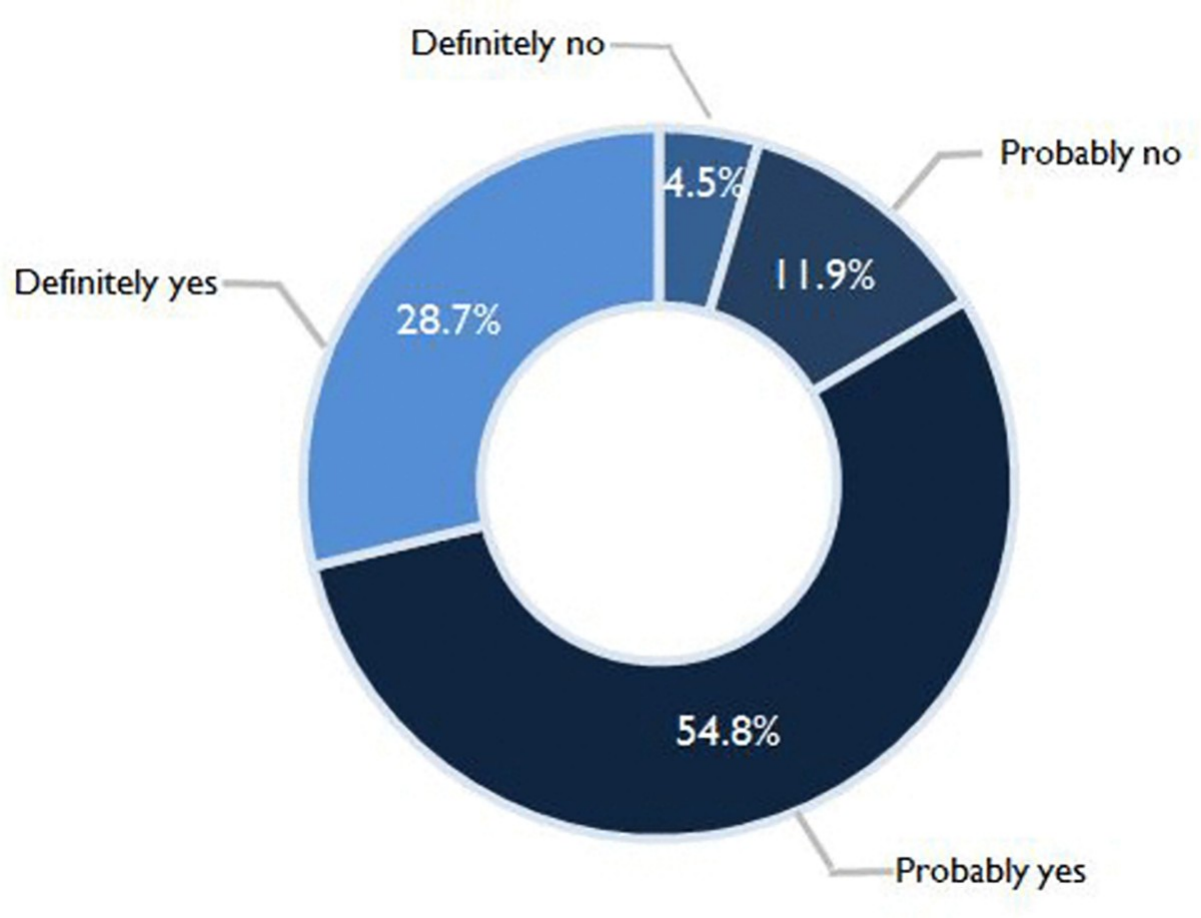
COVID-19 vaccination intent (N = 3541).

Fig 2 shows the proportion of responses for intention to receive a COVID-19 vaccine. On the whole, a total of 2,950 (83.5%; 95%CI 82.3–84.8) participants responded *yes* to COVID-19 vaccine intent, while only 582 (16.4%; 95%CI 15.2–17.7) responded *no*. By a more specific breakdown, the majority responded *probably yes* (54.8%; 95% CI 53.2–56.5) followed by *definitely yes* (28.7%; 95% CI 27.3–30.3%). Only 4.5% (95% CI 3.9–5.3) responded *definitely no* and 11.9% (95% CI 10.8–13.9) reported *probably no*.

The third and fourth columns of Table 2 show the responses of *definitely yes* against the other options (*probably yes/probably no/ definitely no*) in vaccination intention by demographics and health belief constructs. Univariate analyses showed that a significantly higher proportion of participants who were married (30.0%) expressed a definite intention to vaccination than single participants (24.3%); however the association was not significant in the multivariable analysis. By occupational category, a significantly higher proportion that expressed a definite intent to vaccination included people who in a service occupation (35.6%) or who were self-employed (32.7%). There was also significant difference in a definite intention for COVID-19 vaccination by location, whereby participants from the central (41.1%) and southern (32.4%) region reported a higher proportion with a definite intention to receive the COVID-19 vaccine. Participants who perceived their overall health as very good were the highest proportion (34.8%) with a definite intention to vaccinate. By demographics, multivariable analyses revealed that perceived overall health as very good (OR = 1.74, 95% CI 1.44–2.09) and being in the central region of China were strong significant correlates of having a definite intention to vaccinate against COVID-19. Being self-employed (OR = 1.66, 95% CI 1.07–12.59)and in a service occupation (OR = 1.61, 95% CI 1.05–2.49)were also significant correlates of a definite intention to vaccinate.

Most of the constructs in the HBM model were significantly associated with having a definite intention for COVID-19 vaccination in the univariate analysis. Perception that vaccination decreases the chances of getting COVID-19 under the perceived benefit construct (OR = 3.14, 95% CI 2.05–4.83) was the strongest predictor for a definite intention. Being unconcerned about efficacy (OR = 1.65, 95% CI 1.31–2.09) and side effects (OR = 1.52, 95% CI 1.25–1.84) of a new COVID-19 vaccine under the perceived barriers construct were among the strong strongest significant correlates of having a definite intention for COVID-19 vaccination. While the cue to action was a significant construct, participants who disagreed with taking the vaccine unless it had been taken by many in the public were associated with having a higher definite intention to vaccinate (OR = 1.88, 95% CI 1.52–2.33).

### Willingness to pay (WTP)

**Fig 3:**
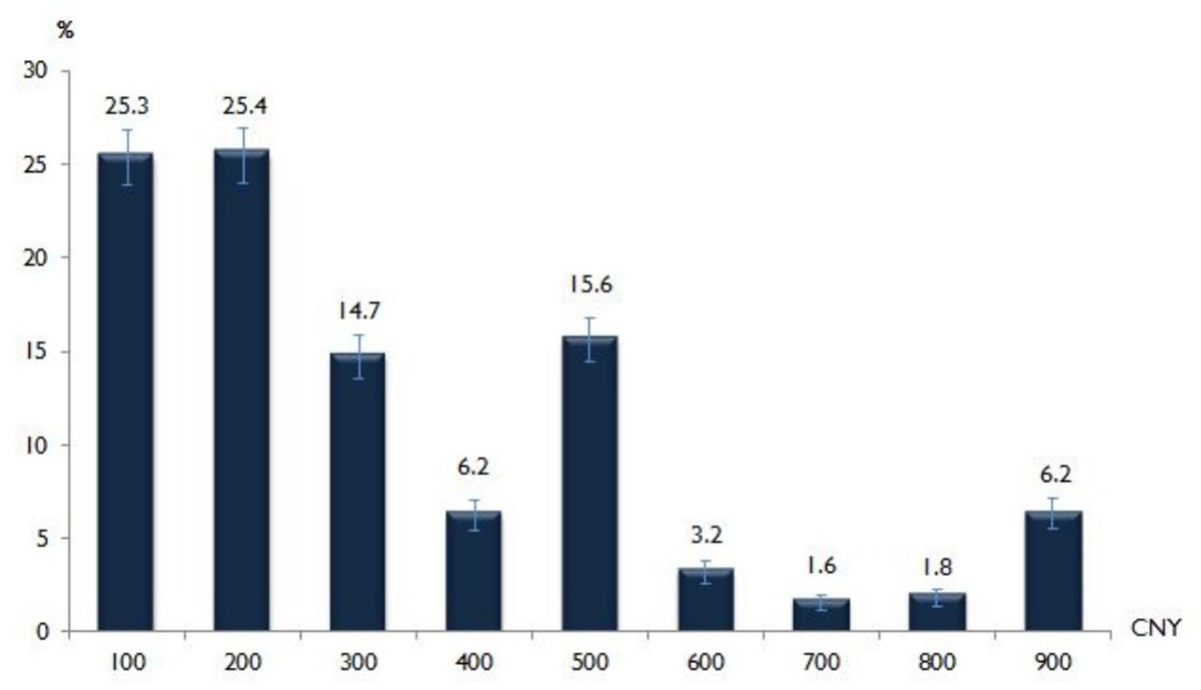
Willingness-to- pay for COVID-19 vaccine (N = 3541).

Fig 3 shows that most of the participants were willing to pay an amount of CNY¥100[US$14] (25.3%, 95% CI 23.9–26.8) or CNY¥200 [US$28] (25.4%, 95% CI 24.0–26.9) for a COVID-19 vaccine. The median (interquartile range [IQR]) of WTP for COVID-19 vaccine was CNY¥200/US$28 (IQR CNY¥100–500/USD$14–72).

Table 3 shows the results of the univariable and multivariable regression analyses for the marginal WTP for an amount of CNY¥100/200[US$14/28], CNY¥300/400 [US$43/57] and ≥CNY¥500 [US$72] by demographics and HBM construct. The results of the multinomial logistic regression (CNY¥300/400 vs. CNY¥100/200 and ≥CNY¥500 vs. CNY¥100/200) revealed that younger aged participants had a higher WTP for an amount of CNY¥300/400 [US$43/57] and ≥CNY¥500 [US$72] over CNY¥100/200[US$14/28]. The professional and managerial group and self-employed participants had the highest WTP for an amount of CNY¥500 [US$72] over CNY¥100/200[US$14/28]. There was a gradual increase in the odds of WTP for an amount of CNY¥500 [US$72] over CNY¥100/200 [US$14/28] with an increase in household income. For HBM constructs, similar to vaccination intention, a higher marginal WTP was significantly associated with the items in the perception of susceptibility and severity of COVID-19, barriers and cues to action constructs.

**Table 3:** Multinomial logic regression of factors associated with marginal willingness to pay (WTP) for COVID-19 vaccine (N = 3541).

	Univariable analysis				Multinomial logistic regression^†^			
	Marginal WTP					Marginal WTP		
	CNY¥100/200 (US$14/28) n = 1798	CNY¥300/400 (US$43/57) n = 734	≥ CNY¥500 (US$72) n = 1006	*p*-value	CNY¥300/400 (US$43/57) OR (95% CI)	*p* value	≥ CNY¥500 (US$72) OR (95% CI)	*p* value
*Demographics*								
Age group (years)								
18–25	294 (45.8)	152 (23.7)	196 (30.5)		1.82 (1.23–2.70)	0.003	3.20 (2.14–4.78)	p<0.001
26–35	790 (47.2)	353 (21.1)	530 (31.7)		1.51 (1.08–2.10)	0.016	2.53 (1.78–3.59)	p<0.001
36–45	466 (54.2)	168 (19.5)	226 (26.3)	p<0.001	1.20 (0.84–1.70)	0.313	1.68 (1.17–2.41)	0.005
46–70	247 (67.5)	65 (17.8)	54 (14.8)		Reference		Reference	
Gender								
Male	858 (50.4	372 (21.9)	472 (27.7)	0.329				
Female	939 (51.1)	366 (19.9)	534 (29.0)					
Marital status								
Married	1428 (51.6)	569 (20.5)	773 (27.9)	0.191				
Single	369 (47.9)	169 (21.9)	233 (30.2)					
Highest education level								
High school and below	718 (57.5)	254 (20.4)	276 (22.1)	p<0.001	1.11 (0.89–1.37)	0370	0.99 (0.80–1.22)	0.914
Tertiary	1079 (47.1)	484 (21.1)	730 (31.8)		Reference		Reference	
Occupation category								
Professionals and managerial	689 (43.3)	354 (22.3)	548 (34.4)		1.28 (0.82–2.01)	0.275	1.76 (1.14–2.74)*	0.012
Industrial workers	282 (66.7)	66 (15.6)	75 (17.7)		0.75 (0.45–1.25)	0.267	0.92 (0.55–1.53)	0.740
Self-employed	181 (43.5)	106 (25.5)	129 (31.0)		1.38 (0.84–2.26)	0.206	1.73 (1.07–2.82)*	0.027
Farmers	77 (60.6)	17 (13.4)	33 (26.0)		0.55 (0.27–1.10)	0.091	1.39 (0.75–2.59)	0.292
Service personnel	218 (52.8)	81 (19.6)	114 (27.6)	p<0.001	1.13 (0.69–1.85)	0.634	1.57 (0.97–2.53)	0.067
Housewife/Retiree/Unemployed	233 (63.3)	71 (19.3)	64 (17.4)		1.03 (0.62–1.72)	0.903	1.09 (0.65–1.82)	0.759
Student	117 (57.6)	43 (21.2)	43 (21.2)		Reference		Reference	
Average annual household income (CNY¥)								
<50000	590 (70.2)	122 (14.5)	129 (15.3)		Reference		Reference	
50001–120000	712 (53.1)	304 (22.7)	325 (24.2)		1.87 (1.46–2.40)	p<0.001	4.19 (3.16–5.56)	p<0.001
120001–170000	251 (38.9)	172 (26.6)	223 (34.5)	p<0.001	2.65 (1.96–3.57)	p<0.001	2.74 (2.06–3.65)	p<0.001
>170000	244 (34.2)	140 (19.6)	329 (46.1)		2.31 (1.69–3.16)	p<0.001	4.19 (3.16–5.56)	p<0.001
Location								
Urban	1491 (49.7)	625 (20.9)	881 (29.4)	0.005	0.93 (0.71–1.20)	0.562	1.10 (0.85–1.43)	0.475
Rural	306 (56.2)	113 (20.8)	125 (23.0)		Reference		Reference	
Current location								
East China	594 (49.9)	233 (19.6)	364 (30.6)		1.11 (0.78–1.58)	0.558	1.23 (0.88–1.72)	0.225
South China	235 (56.4)	78 (18.7)	104 (24.9)	0.001	0.90 (0.60–1.36)	0.625	0.86 (0.58–1.27)	0.436
West China	90 (54.9)	37 (22.6)	37 (22.6)		1.30 (0.79–2.15)	0.302	1.05 (0.63–1.74)	0.860
North China	694 (47.5)	333 (22.8)	433 (29.7)		1.17 (0.83–1.65)	0.380	1.09 (0.78–1.52)	0.619
Central China	184 (59.5)	57 (18.4)	68 (22.0)		Reference		Reference	
*Experience with COVID-19*								
Ever have experience with COVID-19								
No	1690 (51.2)	675 (20.4)	936 (28.4)	0.061				
Yes	107 (44.6)	63 (26.2)	70 (29.2)					
*Health characteristics*								
Ever diagnosed with chronic diseases								
Yes	121 (55.0)	43 (19.5)	56 (25.5)	0.418				
No	1676 (50.5)	695 (20.9)	950 (28.6)					
Perceived overall health								
Very good	567 (49.6)	215 (18.8)	360 (31.5)		0.81 (0.65–1.01)	0.057	1.25 (1.02–1.54)	0.031
Good	569 (50.3)	233 (20.6)	330 (29.2)	0.004	0.89 (0.72–1.10)	0.291	1.17 (0.95–1.43)	0.146
Fair/Poor/Very poor	661 (52.2)	290 (22.9)	316 (24.9)		Reference		Reference	
***Health belief***								
**Perceived susceptibility**								
My chance of getting COVID-19 in the next few months is great								
Strongly agree/agree	519 (46.9)	259 (23.4)	329 (29.7)	0.005	0.97 (0.75–1.24)	0.797	0.95 (0.75–1.20)	0.649
Disagree/strongly disagree	1278 (52.5)	479 (19.7)	677 (27.8)		Reference		Reference	
Worry about the likelihood of getting COVID- 19								
Strongly agree/agree	725 (47.4)	361 (23.6)	442 (28.9)	p<0.001	1.30 (1.04–1.63)*	0.019	1.01 (0.82–1.25)	0.933
Disagree/strongly disagree	1072 (53.3)	377 (18.7)	564 (28.0)		Reference		Reference	
Getting COVID-19 is currently a possibility for me								
Strongly agree/agree	441 (44.5)	237 (23.9)	312 (31.5)	p<0.001	1.21 (0.93–1.56)	0.153	1.33 (1.04–1.69)	0.024
Disagree/strongly disagree	1356 (53.2)	501 (19.6)	694 (27.2)		Reference		Reference	
**Perceived severity**								
Complications from COVID-19 are serious								
Strongly agree/agree	1582 (51.6)	634 (20.7)	849 (27.7)	0.021	1.03 (0.76–1.40)	0.844	0.77 (0.58–1.02)	0.064
Disagree/strongly disagree	215 (45.2)	104 (21.8)	157 (33.0)		Reference		Reference	
I will be very sick if I get COVID-19								
Strongly agree/agree	1073 (49.4)	450 (20.7)	648 (29.8)		1.07 (0.87–1.32)	0.522	1.46 (1.20–1.78)	p<0.001
Disagree/strongly disagree	724 (52.8)	288 (21.0)	358 (26.1)	0.048	Reference		Reference	
I am afraid of getting COVID-19								
Strongly agree/agree	1534 (51.4)	601 (20.1)	849 (28.5)		0.82 (0.61–1.09)	0.172	1.12 (0.84–1.48)	0.449
Disagree/strongly disagree	263 (47.2)	137 (24.6)	157 (28.2)	0.047	Reference		Reference	
**Perceived benefits**								
Vaccination is a good idea because it makes me feel less worried about catching COVID-19								
Strongly agree/agree	1581 (51.2)	625 (20.2)	883 (28.6)					
Disagree/strongly disagree	216 (47.8)	113 (25.0)	123 (27.2	0.065				
Vaccination decreases my chance of getting COVID-19 or its complications								
Strongly agree/agree	1689 (51.5)	659 (20.1)	929 (28.3)	p<0.001	Reference		Reference	
Disagree/strongly disagree	108 (40.9)	79 (29.9)	77 (29.2)		1.48 (1.03–2.13)	0.035	0.91 (0.63–1.32)	0.606
**Perceived barriers**								
Worry the possible side-effects of COVID-19 vaccination would interfere with my usual activities								
Strongly agree/agree	1252 (51.6)	517 (21.3)	656 (27.1)	0.030	1.05 (0.84–1.30)	0.680	0.89 (0.73–1.09)	0.261
Disagree/strongly disagree	545 (48.8)	221 (19.8)	350 (31.4)		Reference		Reference	
Concern about the efficacy of the COVID-19 vaccination								
Strongly agree/agree	1302 (51.7)	517 (20.5)	701 (27.8)	0.226				
Disagree/strongly disagree	495 (48.5)	221 (21.6)	305 (29.9)					
Concern about the safety of the COVID-19 vaccination								
Strongly agree/agree	1323 (51.5)	518 (20.2)	728 (28.3)	0.210				
Disagree/strongly disagree	474 (48.8)	220 (22.6)	278 (28.6)					
Concern of my affordability (high cost) of getting the COVID-19 vaccination								
Strongly agree/agree	1457 (54.8)	549 (20.7)	652 (24.5)	p<0.001	Reference		Reference	
Disagree/strongly disagree	340 (38.5)	189 (21.4)	354 (40.1)		1.34 (1.06–1.71)	0.016	1.97 (1.59–2.45)	p<0.001
Concern if the faulty/fake COVID-19 vaccine								
Strongly agree/agree	1510 (52.0)	598 (20.6)	794 (27.4)	0.003	1.00 (0.77–1.31)	0.997	0.99 (0.78–1.26)	0.938
Disagree/strongly disagree	287 (44.9)	140 (21.9)	212 (33.2)		Reference		Reference	
**Cues to action**								
I will only take the COVID-19 vaccine if I was given adequate information about it								
Strongly agree/agree	1673 (51.3)	671 (20.6)	916 (28.1)	0.068				
Disagree/strongly disagree	124 (44.1)	67 (23.8)	90 (32.0)					
I will only take the COVID-19 vaccine if the vaccine is taken by many in the public								
Strongly agree/agree	1511 (51.9)	610 (20.9)	793 (27.2)	0.002	Reference		Reference	
Disagree/strongly disagree	286 (45.6)	128 (20.4)	213 (34.0)		1.04 (0.80–1.36)	1.043	1.39 (1.10–1.76)	0.006

^†^Multinomial regression; Reference group: CNY¥100/200.

Goodness of fit; Pearson Chi square: 6472.425, Significant: 0.097.

The results of the moderating effect of income level on the relationship between the constructs of HBM and WTP are shown in Table 4. A significant positive moderating effect of income level on the relationship perceived susceptibility and WTP was found (β = 3.007, *p* = 0.045). Income level was also found significantly moderated the relationship between perceived severity and WTP (β = 3.980, *p* = 0.026). This moderation effect of income level was not significant for the relationship between perceived benefit (β = 5.597, *p* = 0.064) and WTP. The level of income also had no moderating effect on the relationship between barriers (β = 0.743, *p* = 0.551) and WTP. Income level showed a strong statistically significant moderating effect on the relationship between cues to action (β = 0.313, *p* = 0.551) and WTP.

**Table 4:** Results of the moderating effect of income level on the relationship between the construct of HBM and WTP.

Source (interaction)	β	SE	*t*	*p*	LLCI	ULCI
Income → susceptibility	3.007*	1.503	2.002	0.045	0.062	5.953
Income → severity	3.980*	1.787	2.227	0.026	0.477	7.484
Income → benefit	5.597	3.017	1.855	0.064	-0.318	11.512
Income → barrier	0.743	1.247	0.596	0.551	-1.702	3.187
Income → cues to action	7.313*	3.076	2.378	0.018	1.282	13.344

*Significant at 0.05 level.

**Fig 4:**
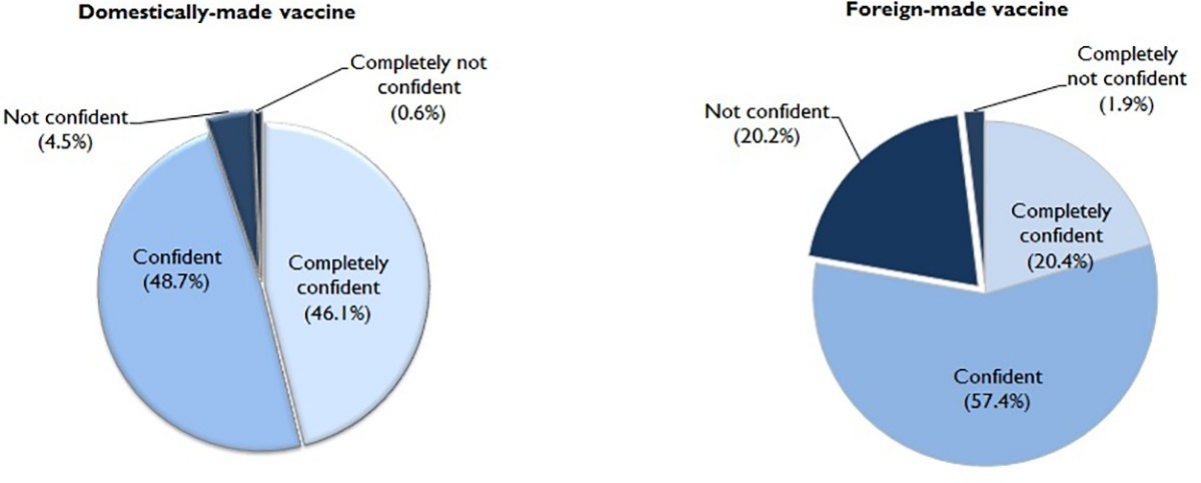
Confidence in domestic-and foreign-made COVID-19 vaccines (N = 3541).

Fig 4 shows the findings on the confidence of domestic and foreign-made COVID-19 vaccines. The vast majority of the study participants reported that they were confident (48.7%, 95%CI 47.0 to 50.3) or completely confident (46.1%, 95%CI 44.5 to 47.8) in domestically-made COVID-19 vaccines. In contrast, 20.4% (95%CI 19.1 to 21.8) reported that they were completely confident and 57.4% (95%CI 55.8 to 59.1) reported that they were confident in foreign-made vaccines. Findings on the preference of domestically-made over foreign-made COVID-19 vaccines revealed that 64.2% (95%CI 62.6 to 65.8) reported a preference for domestically-made and 11.9% (95%CI 10.8 to 13.0) preferred foreign-made COVID-19 vaccines. A total of 23.9% (95%CI 22.5 to 25.4) reported no preference. The summary of key findings is shown in Box 1.

**Box 1. Summary of key findings**

- 83.5% reported vaccination intent, of which only 28.7% stated a definite intent
- Perceived benefits have a strong and positive effect on vaccination intention
- Concerns of side-effects and efficacy were perception of barriers that negatively influence on vaccination intention
- Knowing the vaccine has been taken by many in the public may serve as a cue to action for vaccination intent.
- Median WTP for COVID-19 vaccine was CNY¥200 [US$28]
- The higher marginal WTP for the vaccine was associated with higher socio-economic factors
- Majority expressed confidence in domestically-made COVID-19 vaccine
- Near two-thirds reported a preference for a domestically-made over foreign-made COVID-19 vaccine

## Discussion

This study examined the applicability of the HBM to investigate the intention to receive the COVID-19 vaccine and WTP. The study was conducted after China had declared that the coronavirus outbreak was largely under control. During the data collection period, May 1–19 2020, the total daily confirmed cases of COVID-19 in China were mainly in the single digits, except on 9 and 10 May, where the total number of cases were 14 and 17, respectively. Of notable importance, our data represent a low level of perceived susceptibility of COVID-19 among the study population, thus warranting public enlightenment in terms of not underestimating the possibility of outbreak resurgence, although the country has successfully flattened the infection curve. Previous research implies that high perceived susceptibility and high-risk perception translate into better preventive actions and are associated with enhanced epidemic control [25]. Thus, sustainable preventive and control measures should be encouraged.

In this study, despite a large proportion (83.5%) expressing their intention to vaccinate, only about 30% reported a definite intention. A study from Malaysia, a Southeast Asia country, with confirmed COVID-19 cases of only over four thousand and less than 100 deaths during the study period in April 2020, found a higher (94.3%) vaccination intention, of which 48.2% reported a definite intent [26]. A smaller-scale study in US, which was conducted in May 2020, during which the confirmed cases have passed one million and over 100 thousand deaths reported nevertheless reported a lower (67%) vaccination intent [27,28]. Inter-country comparison of vaccination intention rates by the severity level of the pandemic’s impact is unable to be drawn based on the limited available evidence at present.

A high proportion of a definite intention among participants from the central and southern regions corresponds with high numbers of confirmed cases, indicating that people from regions badly impacted by the coronavirus expressed a higher vaccination intention. Participants in the service occupation expressed higher vaccination intentions, showing their awareness of the need for protection among employees in contact-intensive industries. The fact that China has the world's largest population and being the world’s second-largest economy, a massive surge in demand for the COVID-19 vaccine is anticipated once the vaccine is available. With the intense demand, the COVID-19 vaccination should be prioritized for front-line healthcare workers and vulnerable individuals [28]. Multivariable analysis found that HBM constructs were associated with vaccination intention, which is in concordance with many other studies [29]. In particular, the findings of this study suggest that a high perception of benefits and low perceived barriers to receiving the vaccine were the two most important constructs influencing a definite intention for COVID-19 vaccination. Hence, public health intervention programmes that focus on increasing the perception of the benefits of vaccination and reducing the identified barriers are essential. The perceived barriers against COVID-19 immunizations found in this study, namely worries about side effects and the efficacy of the vaccine, have likewise been reported in other studies related to the introduction of a new vaccine [30]. The identified barriers in this study, namely concerns about efficacy and vaccine adverse events, emphasize that although the search for the COVID-19 vaccine needs to be accelerated, the new vaccine should not bypass established safety and efficacy standards before it is made available to the general public.

China has experienced various negative events associated with vaccine malpractices and scandals that have resulted in public lost confidence in vaccines [31], which perhaps is also implicated in this study as a considerable proportion reported concerns regarding the possibility of fake or faulty COVID-19 vaccines. Regardless of this, a positive finding of this study was that worries about fake or faulty COVID-19 vaccines are not a significant barrier to vaccination intention. The high threat of COVID-19 perhaps overrides concerns about fake or faulty vaccines. Although many were concerned about the cost of the COVID-19 vaccine, the affordability barrier was nonetheless not a significant predictor of definite vaccination intention.

It is important to highlight that the perceptions of the severity of COVID-19 and the perceived benefits in obtaining the COVID-19 vaccine were also significant predictors of a definite intention for COVID-19 vaccination; however, perception of susceptibility was not a significant predictor. External cues to action were found to be important, namely the provision of comprehensive information about the vaccine when it is available. Hence, imparting adequate information to the public is important, particularly the provision of strong evidence of the safety and efficacy of the vaccine from field trials. This study found that slightly over half reported that they would only take the COVID-19 vaccine if the vaccine is taken by many in the public. Further, participants who noted that they will only take the COVID-19 vaccine if it is taken by many in the public expressed lower vaccination intention. The findings imply that promoting COVID-19 vaccination in the forms of advertorials and testimonials may serve as a cue to action to get vaccinated.

This study revealed that most were willing to pay an amount of CNY¥100 [US$14] and CNY¥200 [US$28] for COVID-19 vaccine. Although the median WTP of the overall participants was CNY¥200 [US$28], it is important to note that the public from many parts of the country may not be able to afford the vaccine at this price. China’s income inequality ranks among the highest in the world and it is largely due to regional disparities and the urban-rural gap [32,33]. Furthermore, this study also found that the highest marginal WTP for the vaccine was influenced by socio-economic factors and younger aged people expressed/reported higher marginal WTP. Owing to the nationwide economic disruption resulting from the COVID-19 pandemic, it is crucial that people of income levels can access the vaccines without imposing an additional financial burden. To ensure population-wide access, the coronavirus vaccine should be provided free of charge or at a subsidized rate to low-income individuals. In China, obligatory vaccines or Category 1 vaccines are made available through the government’s Expanded Program on Immunization (EPI) at no charge for children up to 14 years old [8,9].The Category 2 vaccines, including the influenza vaccines, must be purchased by the public, and thus have a price barrier [34]. The WTP findings from this study could serve as a useful reference in directing China’s future vaccine pricing and procurement systems. Perceived susceptibility and severity of COVID-19 correlated positively with higher marginal WTP, while the perceived economic barrier was negatively correlated with higher marginal WTP. Thus, it is necessary to provide information based on the HBM to enhance the public’s willingness to pay for the vaccine when it available. Measures to reduce the level of out-of-pocket payment across the lower socioeconomic groups are also important. Of important note, the significant positive moderating effect of income on the relationship between the health beliefs (perceived susceptibility, severity, and cues to action) and WTP suggests greater levels of relationship at higher levels of income. The findings suggest that HBM-base intervention would be useful for the higher-income groups to enhance their WTP for the COVID-19 vaccine.

Another positive highlight of this study is the high confidence of the study participants in domestically-made COVID-19 vaccines, and a higher preference for domestically-made than foreign-made COVID-19 vaccines. The new Vaccine Administration Law of the People’s Republic of China, effective December 1 2019, has greatly enhanced regulations in the vaccine industry. The new law stipulates stringent regulatory requirements for researching, producing, distributing, and using vaccines. The new law also imposes tough penalties for violating regulations on the production and sales of vaccines [35]. The implementation of China’s new vaccine administration law has perhaps boosted the public’s confidence and increased public trust in domestic vaccines. The summary of recommendations for health authorities and messages to the communities is shown in Box 2.

**Box 2. Recommendations for health authorities and messages to communities**

- Efforts are needed to promote high definite intention to get vaccinated against COVID-19 when the vaccine is successfully developed and given regulatory approval
- Public health intervention programmes should focus on increasing the perception of the benefits of COVID-19 vaccination and reducing the perceived adverse effect and inefficacy barriers
- Clinical evidence of the safety and efficacy of COVID-19 vaccines are key messages to enhance rates of vaccine coverage
- Promoting COVID-19 vaccination in the forms of advertorials and testimonials may prompt vaccination decision
- It is crucial to reduce inequalities in access to COVID-19 vaccines due to financial constraints
- Further measures are needed to identify and restore confidence in domestically-made COVID-19 vaccines among certain individuals and societal groups

There are some limitations of the current study that need to be considered in interpreting the results. Firstly, the use of an online survey may result in sampling bias, so results may not be generalizable to the wider community as reflected in lack of representative from some provinces. The intrinsic disadvantages of Web-based surveys concern the generality and validity of results warranting careful interpretation of the research findings [36,37]. It is notable adults between the ages of 26 and 35 are over-represented in this survey sample, while older adults ages 46–70 are under-represented. The use of WeChat to advertise and circulate the survey link to network members may result in selection bias due to the non-representative nature of the contact network population. Furthermore, the responses were based on self-report and may be subject to self-reporting bias and a tendency to report socially desirable responses. It is also noteworthy that the assessment of intention to vaccinate in this study did not account for the other possible factors affecting vaccination intention such as duration of protection of the new vaccine and the need for booster doses which may have a significant influence on participants' intention. Another limitation of the study is the bias associated with the assessment of acceptance and WTP for a hypothetical COVID-19 vaccine before the final product exists [38]. The assessment of vaccine acceptance and WTP in real contexts would reflect consumers’ actual acceptance and WTP. Therefore, there is a need for additional research to gather accurate data about COVID-19 vaccine acceptance and WTP when the vaccine is successfully developed and available to the public. Additionally, future studies should also consider examining the acceptance and WTP in the context of other epidemiologically important parameters such as different levels of community transmission of COVID-19. In this study, although the COVID-19 vaccine acceptance is higher among respondents from central China, the epicenter of the outbreak, owing to the limitation in the study design, further methodologically sound studies are warranted to conclusively determine vaccination acceptance in different community spread areas in China. This information is especially important to provide insights into targeted intervention for improving vaccine coverage, particularly in high virus circulating settings. The findings of this survey should be interpreted in light of the above-mentioned limitations. Despite these limitations, we believe that our findings contribute tremendously to understanding public demand and hesitancy regarding the COVID-19 vaccine.

## Conclusion

Health promotion is warranted when the COVID-19 vaccine is available because, among those who expressed intention to be vaccinated, over half expressed probable intention. The HBM model can be used to develop strategies for enhancing COVID-19 vaccine uptake and WTP. Most importantly, the findings revealed that interventions targeting perceived barriers, i.e. the efficacy and adverse events related to a new COVID-19 vaccine, are most crucial. Concerns regarding faulty or fake vaccines were not a significant predictor for COVID-19 vaccination intent, perhaps implying public trust in the government’s new vaccine administration law.The WTP was positively related to socio-economic factors, which should provide guidance for policy recommendations for the future national COVID-19 improvisation program in mainland China.

## Supporting information

S1 Text Full questionnaire. (DOCX)
