# Supplementary material for: Understanding COVID-19 vaccine demand and hesitancy: A nationwide online survey in China
Source: PLoS Negl Trop Dis. 2020 Dec 17;14(12):e0008961. doi: 10.1371/journal.pntd.0008961 (PMC7775119; doi:10.1371/journal.pntd.0008961)
Supplement: S1 Text — (DOCX) [file pntd.0008961.s001.docx]

**FULL QUESTIONNAIRE**

**Acceptance, attitudes, and willingness to pay for a future COVID-19 vaccination**

Section A

**GENERAL INFORMATION**

| 1 | Age | _______________________________years old |
| --- | --- | --- |
| 2 | Gender | [ 1 ] Male  [ 2 ] Female |
| 3 | Ethnicity | [ 1 ] Han  [ 2 ] Others |
| 4 | Marital status | [ 1 ] Married  [ 2 ] Single |
| 5 | Highest education level | [ 1 ] Primary school and below  [ 2 ] Secondary school  [ 3 ] High school  [ 4 ] University and above |
| 6 | Occupation | [ 1 ] Industrial workers  [ 2 ] Farmers  [ 3 ] Self-employed  [ 4 ] Professional worker (healthcare provider, teacher ,lawyer)  [ 5 ] Manager in company, industry, ect.  [ 6 ] Officer in government, office  [ 7 ] Service personnel  [ 8 ] House wife  [ 9 ] Retired  [ 10 ] Unemployed  [ 11 ] Students  [ 12 ] Others |
| 7 | Annually average household income (RMB) | [ 1 ] <50000  [ 2 ] 50001-120000  [ 3 ] 120001-170000  [ 4 ] >170000  [ 5 ] ≥2500001 |
| 8 | Residence area | [ 1 ] Urban  [ 2 ] Rural |
| 9 | Current location   \|  \| \| --- \| \|  \| \|  \| \|  \| | _________________________province  _________________________city |
| COVID-19 experience | | |
| 10 | Ever have experience with COVID-19 | [ 1 ] Yes  [ 2 ] No |
| General health | | |
| 11 | Do you have an existing chronic disease such as cancer, cardiovascular disease, diabetes, etc | [ 1 ] Yes  [ 2 ] No |
| 12 | How do you rate your overall health | [ 1 ] Very good  [ 2 ] Good  [ 3 ] Fair  [ 4 ] Poor  [ 5 ] Very poor |

Section B

**Acceptability of COVID-19 vaccine if available in the market**

| 1 | If vaccine against COVID-19 infection is available in the market, would you take it? | **[** ] Definitely no  [ ] Probably no  [ ] Possibly yes  [ ] Definitely no |
| --- | --- | --- |

Section C

**Preference of COVID-19 vaccine**

| 1 | Rate your **confidence in using** local manufactured COVID-19 vaccine | [ 1 ] Completely confident  [ 2 ] Confident  [ 3 ] Not confident  [ 4 ] Completely not confident |
| --- | --- | --- |
| 2 | Rate your **confidence in using** **foreign** manufactured (imported) COVID-19 vaccine | [ 1 ] Completely confident  [ 2 ] Confident  [ 3 ] Not confident  [ 4 ] Completely not confident |
| 3 | Please indicate your preferences in local/imported COVID-19 vaccine | ONLY TICK ONE  [ 1 ] I prefer local manufactured COVID-19 vaccine.  [ 2 ] I prefer imported/foreign manufactured COVID-19 vaccine.  [ 3 ] I don’t have preference as long as there is vaccine available |

Section D

**Health Belief Model**

| ***Perceived susceptibility of contracting COVID-19*** | | |
| --- | --- | --- |
| 1 | My chance of getting COVID-19 in the next few months is great | [ 1 ] Strong agree  [ 2 ] Agree  [ 3 ] Disagree  [ 4 ] Strongly Disagree |
| 2 | I am worried about the likelihood of getting COVID 19 | [ 1 ] Strong agree  [ 2 ] Agree  [ 3 ] Disagree  [ 4 ] Strongly Disagree |
| 3 | Getting COVID-19 is currently a possibility for me. | [ 1 ] Strong agree  [ 2 ] Agree  [ 3 ] Disagree  [ 4 ] Strongly Disagree |
| ***Perceived Severity*** | | |
| 4 | Complications from COVID-19 are serious | [ 1 ] Strong agree  [ 2 ] Agree  [ 3 ] Disagree  [ 4 ] Strongly Disagree |
| 5 | I will be very sick if I get COVID-19 | [ 1 ] Strong agree  [ 2 ] Agree  [ 3 ] Disagree  [ 4 ] Strongly Disagree |
| 6 | I am afraid of getting COVID-19 | [ 1 ] Strong agree  [ 2 ] Agree  [ 3 ] Disagree  [ 4 ] Strongly Disagree |
| ***Perceived benefits of COVID-19 vaccination*** | | |
| 7 | Vaccination is a good idea because I feel less worried about catching COVID-19 | [ 1 ] Strong agree  [ 2 ] Agree  [ 3 ] Disagree  [ 4 ] Strongly Disagree |
| 8 | Vaccination decreases my chance of getting COVID-19 or its complications | [ 1 ] Strong agree  [ 2 ] Agree  [ 3 ] Disagree  [ 4 ] Strongly Disagree |
| ***Perceived barriers of COVID-19 vaccination*** | | |
| 9 | Worry the possible ***side-effects*** of COVID-19 vaccination would interfere with my usual activities | [ 1 ] Strong agree  [ 2 ] Agree  [ 3 ] Disagree  [ 4 ] Strongly Disagree |
| 10 | I am concern about the ***efficacy*** of the COVID-19 vaccination | [ 1 ] Strong agree  [ 2 ] Agree  [ 3 ] Disagree  [ 4 ] Strongly Disagree |
| 11 | I am concern about the ***safety*** of the COVID-19 vaccination | [ 1 ] Strong agree  [ 2 ] Agree  [ 3 ] Disagree  [ 4 ] Strongly Disagree |
| 12 | I am concern of my ***affordability (high cost of the vaccine)*** of getting the COVID-19 vaccination | [ 1 ] Strong agree  [ 2 ] Agree  [ 3 ] Disagree  [ 4 ] Strongly Disagree |
| 13 | I am concern of the faulty/fake COVID-19 vaccine | [ 1 ] Strong agree  [ 2 ] Agree  [ 3 ] Disagree  [ 4 ] Strongly Disagree |
| ***Cues to action*** | | |
| 14 | I will only take the COVID-19 vaccine if I was given adequate information about it | [ 1 ] Strong agree  [ 2 ] Agree  [ 3 ] Disagree  [ 4 ] Strongly Disagree |
| 15 | I will only take the COVID-19 vaccine if the vaccine is taken by many in the public | [ 1 ] Strong agree  [ 2 ] Agree  [ 3 ] Disagree  [ 4 ] Strongly Disagree |

Section E

**Willingness to Pay**

| 1 | What is the maximum amount you are willing to pay the COVID-19 vaccine per dose for your own vaccination? | [ 1 ] RMB100  [ 2 ] RMB200  [ 3 ] RMB300  [ 4 ] RMB400  [ 5 ] RMB500  [ 6 ] RMB600  [ 7 ] RMB700  [ 8 ] RMB800  [ 9 ] RMB900 |
| --- | --- | --- |
